# Hemoglobin‐platelet index as a prognostic factor in patients with peripheral T‐cell lymphoma

**DOI:** 10.1002/jha2.727

**Published:** 2023-05-26

**Authors:** Yu Yagi, Yusuke Kanemasa, Yuki Sasaki, Shunichi Okumura, Takako Watanabe, Kento Ishimine, Yudai Hayashi, Mano Mino, An Ohigashi, Yuka Morita, Taichi Tamura, Shohei Nakamura, Toshihiro Okuya, Tatsu Shimoyama

**Affiliations:** ^1^ Department of Medical Oncology Tokyo Metropolitan Cancer and Infectious Diseases Center Komagome Hospital Tokyo Japan; ^2^ Department of Pharmacy Tokyo Metropolitan Cancer and Infectious Diseases Center Komagome Hospital Tokyo Japan

**Keywords:** albumin, hemoglobin‐platelet index, lactate dehydrogenase, peripheral T‐cell lymphoma, prognostic factor, survival

## Abstract

Peripheral T‐cell lymphoma (PTCL) is a heterogeneous group of aggressive lymphomas with a poor prognosis. The International Prognostic Index (IPI) and the Prognostic Index for PTCL‐unspecified (PIT) is used to predict the prognosis of PTCL. The hemoglobin‐platelet index (HPI), based on anemia and thrombocytopenia status, is associated with the prognosis of diffuse large B‐cell lymphoma. However, its significance in terms of predicting the prognosis of PTCL has not been fully investigated. We herein retrospectively analyzed 100 patients with newly diagnosed PTCL in our department. At a median follow‐up of 3.2 years, the median progression‐free survival (PFS) and overall survival (OS) was 0.72 (95% confidence interval [CI]: 0.56–1.2) years and 2.0 (95% CI: 1.5–4.7) years, respectively. Multivariate analysis revealed that elevated lactic dehydrogenase (LDH) and hypoalbuminemia were independent adverse variables for PFS. The HPI showed significant predictive value for both PFS and OS. As a new prognostic model comprising the HPI, LDH, and albumin, the LA‐HPI allowed the stratification of patients into four distinct risk subgroups: low risk (zero risk factors), low‐intermediate risk (one risk factors), high‐intermediate risk (two or three risk factors), or high risk (four risk factors). The PFS and OS differed significantly among the patients by the LA‐HPI score. The LA‐HPI demonstrated better predictive performance compared to the IPI, PIT, and HPI. Our data demonstrated the prognostic utility of the HPI in patients with PTCL. The LA‐HPI, incorporating four readily obtainable parameters, exhibited superior performance compared to traditional indices.

## 1 INTRODUCTION

Peripheral T‐cell lymphoma (PTCL) comprises a heterogeneous group of lymphoid malignancies constituting approximately 10% of non‐Hodgkin's lymphoma cases (NHLs) in adults [1]. The most common subtypes are the so‐called nodal PTCLs, including PTCL‐not otherwise specified (PTCL‐NOS), angioimmunoblastic T‐cell lymphoma (AITL), and systemic anaplastic large cell lymphoma (ALCL) [2]. Although anaplastic lymphoma kinase (ALK)‐positive ALCL has a relatively favorable prognosis in younger patients, older patients or those with a high International Prognostic Index (IPI) score (≥2) have a prognosis similar to that of patients with ALK‐negative ALCL [3]. These subtypes are usually treated with cyclophosphamide, doxorubicin, vincristine, and prednisone (CHOP) or other CHOP‐like regimens [4]. Although a small proportion of patients with PTCL might respond to, and even be cured with, conventional chemotherapy, the outcome of patients with PTCL is generally dismal [5]. Consolidation at first remission with high‐dose chemotherapy and autologous stem cell transplantation (ASCT) is often performed in young and fit patients in the hope of achieving better disease control. Unfortunately, however, most cases of PTCL eventually relapse after ASCT, and the superiority of this strategy has not been established [4, 6, 7]. Brentuximab vedotin (BV), an antibody–drug conjugate targeting CD30 plus cyclophosphamide, doxorubicin, and prednisone (BV+CHP) was approved after a randomized phase 3 trial (ECHELON‐2). This approach resulted in a 5‐year overall survival (OS) and progression‐free survival (PFS) of 70.1% and 51.4%, respectively [8]. Although this novel agent has contributed to better survival outcomes in patients with PTCL, the outcomes are still less than satisfactory, and the search for better treatment options continues urgently.

Many previous studies have described the prognostic factors in the therapeutic response and survival of patients with PTCL. The IPI and the Prognostic Index for PTCL‐unspecified (PIT) are the main instruments for assessing the prognosis of patients with PTCL [9, 10]. Further, the predictive potential of several inflammatory parameters, such as C‐reactive protein (CRP), serum ferritin, and β2‐microglobulin, has been investigated [11, 12, 13], and the Glasgow Prognostic Score (GPS), an inflammation‐based prognostic score based on CRP and albumin values, can also predict the prognosis of PTCL [14]. The controlling nutritional status (CONUT) score, a simplified nutritional index based on serum albumin, total cholesterol, and total lymphocyte values, is also reportedly an independent prognostic factor in patients with PTCL [15].

Several clinical markers using hematological parameters, such as platelet count and hemoglobin (Hb) level, have also been recognized as important prognostic factors for PTCL [16, 17, 18]. The hemoglobin‐platelet index (HPI) is calculated using the Hb value and platelet count, and is also reportedly an independent prognostic factor for diffuse large B‐cell lymphoma (DLBCL) [19]. However, its prognostic value for patients with PTCL has not been specifically investigated.

In this study, we aimed to investigate the prognostic factors associated with PTCL. Furthermore, we explored the predictive value of the HPI and assessed the potential for refinement of this index.

## 2 MATERIALS AND METHODS

### 2.1 Patients

All consecutive patients with PTCL between March 2004 and August 2022 who were treated at our department were included. Lymphoma was pathologically diagnosed in accordance with the World Health Organization (WHO) classification [20, 21]. All pathology specimens were reviewed by expert pathologists at our hospital. Patients meeting the criteria for adult T‐cell leukemia/Lymphoma and natural killer/T‐cell lymphoma were excluded, because these lymphomas are generally treated using a different strategy. None of the patients had previously been treated for PTCL. Clinical staging was performed using the Ann Arbor classification. Performance status (PS) was evaluated using the Eastern Cooperative Oncology Group (ECOG) criteria. The clinical tumor response was assessed using computed tomography (CT) or positron emission tomography‐CT (PET‐CT) according to the International Workshop Criteria or Lugano Criteria [22, 23]. The present study was conducted in accordance with the Declaration of Helsinki, and the protocol was approved by the Ethics Committee of Tokyo Metropolitan Cancer and Infectious Diseases Center at Komagome Hospital, which waived the requirement for consent because only retrospective data obtained from the hospital medical records were used.

### 2.2 Prognostic scores in PTCL

The IPI and PIT scores were calculated as reported previously [9, 10]. The IPI scores were calculated using age, serum lactate dehydrogenase (LDH), PS, Ann Arbor stage, and extranodal involvement at diagnosis. The PIT scores were based on age, PS, LDH, and bone marrow involvement. The HPI was calculated by assigning one point to the presence of anemia (Hb < 13 g/dL for male patients and Hb < 12 g/dL for female patients) or thrombocytopenia (platelets <100 × 10^9^/L). Patients were divided into high‐risk (score 2), intermediate‐risk (score 1), and low‐risk (score 0) groups [19].

### 2.3 Statistical analysis

OS was defined as the time from diagnosis to death or the latest follow‐up. PFS was defined as the time from diagnosis to relapse or death from any cause or the latest follow‐up. OS and PFS were estimated using the Kaplan–Meier method and were compared using univariate analysis with the log‐rank test. Risk factors statistically significant on univariate analysis were included in multivariate analysis. The differences in the characteristics of the two groups were assessed using Fisher's exact test. The discriminatory power and prognostic performance of each prognostic index were assessed using Harrell's C‐index and area under the curve (AUC), which was derived from the receiver operating characteristic (ROC) curve. All *p*‐values were two‐sided, and *p* < 0.05 was considered to indicate statistical significance. All statistical analyses were performed with R software (version 4.2.2) or EZR software (version 1.41) [24].

## 3 RESULTS

### 3.1 Patient characteristics

In total, 100 patients meeting the criteria described above were included. The median follow‐up period in the surviving patients was 3.2 years (range: 0.54–11.6 years). Table 1 summarizes their clinical characteristics. The median age was 70 years (range: 20–91 years). Thirty‐one (31.0%), 43 (43.0%), six (6.0%), 11 (11.0%), and four (4.0%) patients had a diagnosis of PTCL‐NOS, AITL, ALK‐positive ALCL, and ALK‐negative ALCL with enteropathy‐associated T‐cell lymphoma (EATL), respectively. One patient had intestinal T‐cell lymphoma, two patients had cutaneous T‐cell lymphoma, and two patient had follicular T‐cell lymphoma. Most of the patients (*n* = 87; 87.0%) received an anthracycline‐based regimen as their first‐line chemotherapy. Of these, 58 (58.0%) received CHOP, 15 (15.0%) received BV+CHP, eight (8.0%) received THP‐COP, two (2.0%) received HyperCVAD, and four (4.0%) received another CHOP‐like regimen. Among 85 patients whose therapeutic response to anthracycline‐based chemotherapy was able to be assessed, the overall response rate (ORR) was 67.1%, with 44.7% having complete remission and 22.4% having partial remission. After showing a response to the first‐line therapy, one patient received consolidation radiotherapy, and two underwent ASCT. Of the 58 patients with relapsed or refractory disease, four underwent ASCT, and four underwent allogeneic stem cell transplantation.

**TABLE 1:** Baseline patient characteristics

Characteristic	No. (%)
No. of patients	100
Median age at diagnosis (range) in years	70 (20–91)
Sex (male)	62 (62)
B symptoms	52 (52)
Stages III–IV	72 (72)
Bone marrow involvement	14/98 (14.3)
>1 extranodal sites	26 (26)
ECOG PS > 1	27 (27)
LDH > ULN	51 (51.0)
Albumin <3.0 g/dL	28 (28)
CRP (>1.0 mg/dL)	53 (53)
sIL‐2R (>3000 U/mL)	47/99 (47.5)
β2 Microglobulin (>1.9 μg/mL)	69/80 (86.2)
Platelets (<10,000/μL)	9 (9)
Anemia (Hb < 13 g/dL for male patients and Hb < 12 g/dL for female patients)	63 (63)
NLR (>4)	45 (45)
Diagnosis	
PTCL‐NOS	31 (31)
AITL	43 (43)
ALK‐positive ALCL	6 (6)
ALK‐negative ALCL	11 (11)
EATL	4 (4)
Other	5 (5)
IPI	
Low	26 (26)
Low‐intermediate	22 (22)
High‐intermediate	25 (25)
High	27 (27)
PIT	
Group 1	8 (8.3)
Group 2	39 (39.8)
Group 3	33 (33.7)
Group 4	18 (18.4)
Not calculable	2
First‐line chemotherapy	
CHOP	58 (58)
BV+CHP	15 (15)
THP‐COP	8 (8)
Hyper‐CVAD	2 (2)
CHOP‐like	4 (4)
Other	4 (4)
No treatment or steroid alone	9 (9)

Abbreviations: AITL, angioimmunoblastic T‐cell lymphoma; ALCL, anaplastic large‐cell lymphoma; ALK, anaplastic lymphoma kinase; BV+CHP, brentuximab vedotin, cyclophosphamide, doxorubicin, and prednisone; CHOP, cyclophosphamide, doxorubicin, vincristine, and prednisone; CRP, C‐reactive protein; EATL, enteropathy‐associated T‐cell lymphoma; ECOG PS, Eastern Cooperative Oncology Group performance status; Hb, hemoglobin; HyperCVAD, hyperfractionated cyclophosphamide, vincristine, doxorubicin and dexamethasone; IPI, International Prognostic Index; LDH, lactate dehydrogenase; NLR, neutrophil/lymphocyte ratio; PIT, prognostic index for PTCL, unspecified; PTCL‐NOS, peripheral T‐cell lymphoma‐not otherwise specified; sIL‐2R, soluble interleukin‐2 receptor; THP‐COP, tetrahydropyranyl adriamycin, cyclophosphamide, vincristine, and prednisolone; ULN, upper limit of normal.

### 3.2 Outcomes in the entire study cohort

**FIGURE 1:**
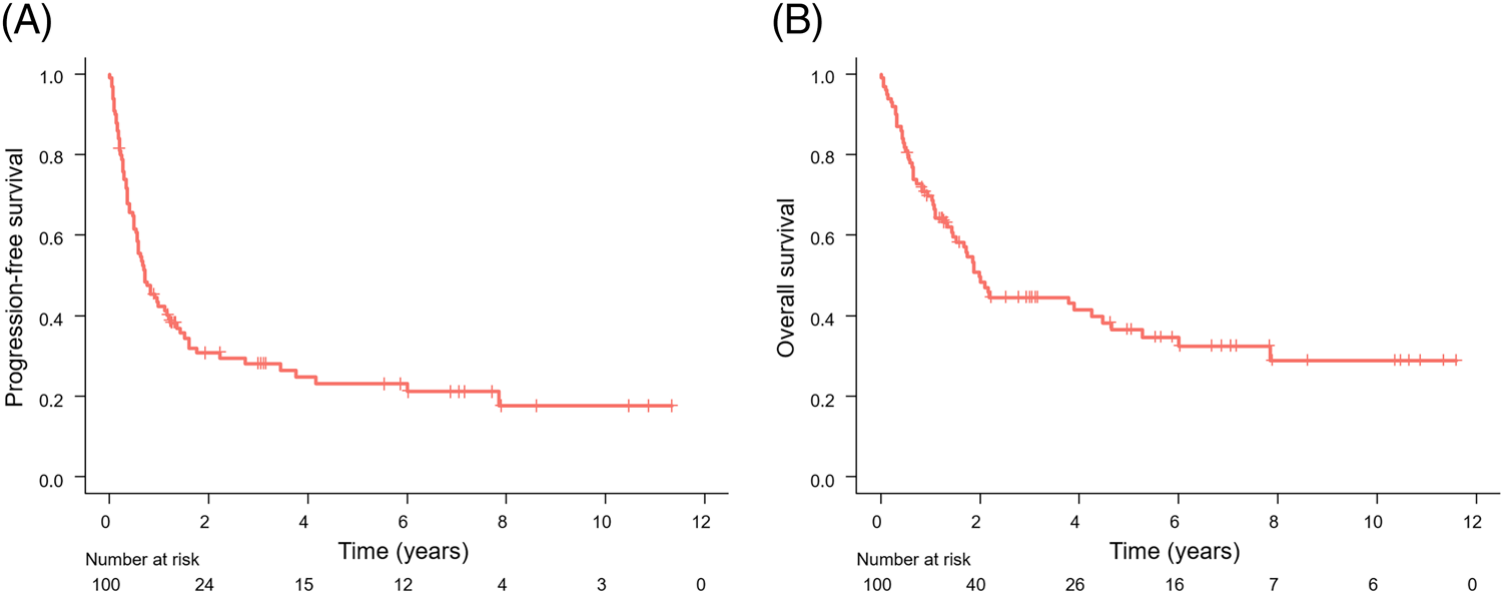
Kaplan–Meier analysis of progression‐free survival (A) and overall survival (B) in the entire cohort.

In the total cohort, 63 patients experienced a relapse or progression of the lymphoma, and 59 patients died. Fifty patients died from lymphoma, two patients died from another malignancy, and one patient died from infection. The causes of death were unknown in the other patients. The median PFS was 0.72 years (95% CI: 0.56–1.2 years), and the 5‐year estimated PFS was 23.2% (95% CI: 15.6%–34.5%) in all the patients (Figure 1A). The median OS was 2.0 years (95% CI: 1.5–4.7 years), and the 5‐year estimated OS was 36.7% (95% CI: 27.4%–49.1%) in all the patients (Figure 1B).

### 3.3 Outcomes by histological subtype

**FIGURE 2:**
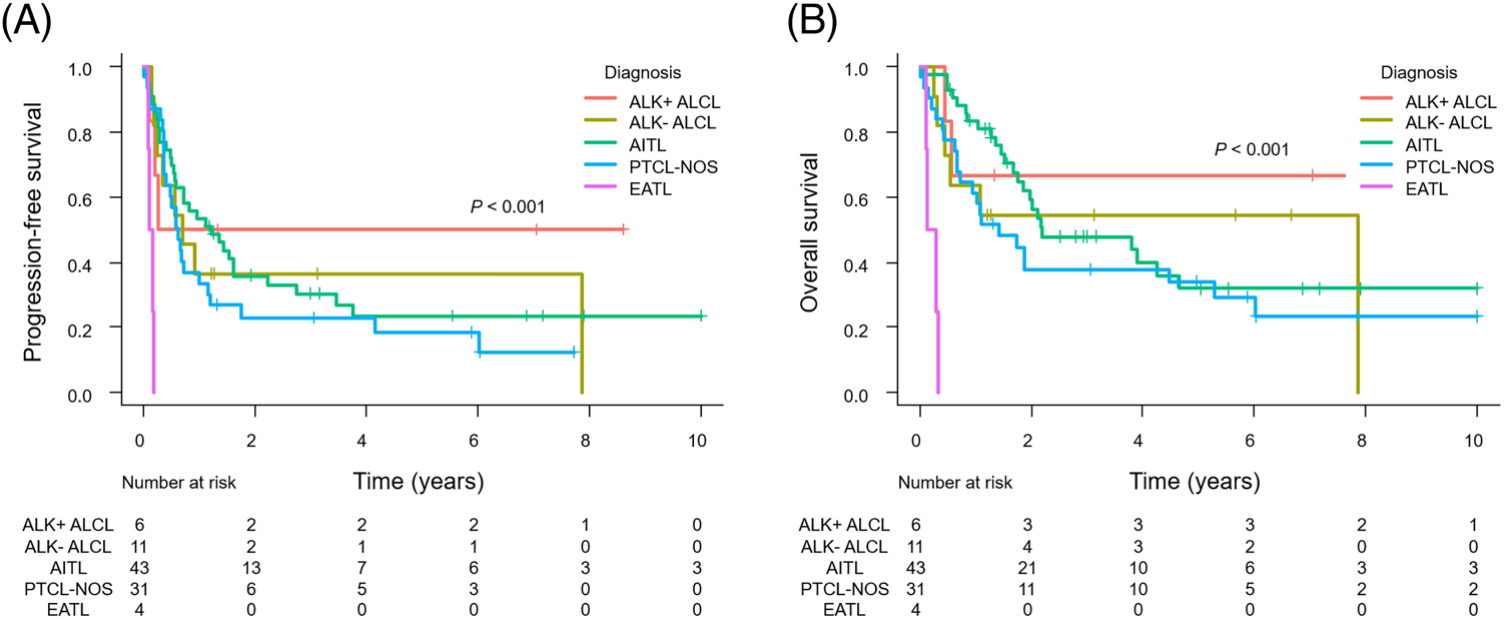
Kaplan–Meier analysis of progression‐free survival (A) and overall survival (B) based on histological subtype.

The 5‐year PFS for the individual groups was as follows: PTCL‐NOS: 18.4%; AITL: 23.6%; ALK‐positive ALCL: 50.0%; ALK‐negative ALCL: 36.4%; and EATL: 0% (Figure 2A). The 5‐year OS for the individual groups was as follows: PTCL‐NOS: 33.6%; AITL: 31.9%; ALK‐positive ALCL: 66.7%; ALK‐negative ALCL: 54.5%; and EATL: 0% (Figure 2B). EATL was the only subtype with a PFS and OS significantly inferior to that of PTCL‐NOS (PFS: median 0.13 vs. 0.62 years; *p* < 0.001; OS: median 0.20 vs. 1.4 years; *p* < 0.001).

### 3.4 Prognostic significance of the clinical factors

Table 2 shows the results of univariable and multivariable analysis of PFS and OS in the entire cohort. On univariate analysis, ECOG PS > 1, extranodal sites greater than one, elevated LDH, hypoalbuminemia, thrombocytopenia, and anemia had a significant negative impact on PFS. Similarly, ECOG PS > 1, elevated LDH, hypoalbuminemia, thrombocytopenia, and anemia were significant adverse factors for OS. On Cox regression analysis, elevated LDH and hypoalbuminemia were independent risk factors of PFS, and hypoalbuminemia was the only independent risk factor of OS.

**TABLE 2:** Univariate and multivariate analyses of clinical factors of progression‐free survival and overall survival

	PFS				OS			
	**Univariate analysis**		**Multivariate analysis**		**Univariate analysis**		**Multivariate analysis**	
Factor	**Hazard ratio** **(95% CI)**	***p***	**Hazard ratio** **(95% CI)**	***p***	**Hazard ratio** **(95% CI)**	***p***	**Hazard ratio** **(95% CI)**	***p***
Age (>60)	1.12 (0.65–1.93)	0.68			1.72 (0.87–3.4)	0.12		
Performance status (>1)	1.89 (1.14–3.13)	0.014	1.14 (0.66–1.97)	0.64	2.41 (1.39–4.18)	0.002	1.53 (0.85–2.76)	0.16
Ann Arbor stage (III–IV)	1.12 (0.45–2.78)	0.81			0.95 (0.35–2.65)	0.94		
Extranodal sites (>1)	1.96 (1.19–3.22)	0.008	1.59 (0.96–2.64)	0.075	1.35 (0.76–2.39)	0.31		
Bone marrow involvement	1.34 (0.72–2.48)	0.36			1.68 (0.89–3.17)	0.11		
LDH > ULN	2.56 (1.59–4.13)	< 0.001	1.77 (1.05–2.99)	0.032	1.97 (1.16–3.34)	0.012	1.19 (0.66–2.14)	0.56
Albumin (≤3.0 g/dL)	2.97 (1.82–4.86)	< 0.001	1.95 (1.11–3.43)	0.020	3.34 (1.93–5.75)	<0.001	2.65 (1.39–5.04)	0.003
Platelets (<10,000/μL)	2.36 (1.17–4.76)	0.017	1.93 (0.93–4.00)	0.077	2.16 (1.02–4.57)	0.043	2.10 (0.95–4.63)	0.068
Anemia (Hb < 13 g/dL for males and Hb < 12 g/dL for females)	1.64 (1.0–2.67)	0.047	1.15 (0.69–1.93)	0.59	1.95 (1.1–3.43)	0.022	1.38 (0.75–2.53)	0.30

Abbreviations: AITL, angioimmunoblastic T‐cell lymphoma; ALCL, anaplastic large‐cell lymphoma; ALK, anaplastic lymphoma kinase; CRP, C‐reactive protein; EATL, enteropathy‐associated T‐cell lymphoma; Hb, hemoglobin; LDH, lactate dehydrogenase; NLR, neutrophil/lymphocyte ratio; OS, overall survival; PFS, progression‐free survival; PTCL‐NOS, peripheral T‐cell lymphoma‐not otherwise specified; sIL‐2R, soluble interleukin‐2 receptor; ULN, upper limit of normal.

### 3.5 Conventional prognostic scores of PTCL

**FIGURE 3:**
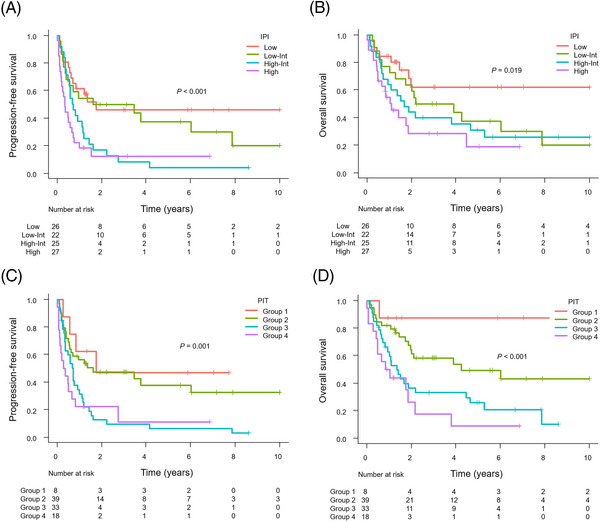
Kaplan–Meier analysis of progression‐free survival (PFS) and overall survival (OS) according to the International Prognostic Index (IPI) and Prognostic Index for PTCL‐unspecified (PIT). (A) PFS according to the IPI. (B) OS according to the IPI. (C) PFS according to the PIT. (D) OS according to the PIT.

The IPI and PIT significantly stratified PFS and OS (Figure 3). However, the differentiation of survival curves was suboptimal in some areas; for example, the IPI failed to demonstrate a significant distinction in PFS between the low‐risk and low‐intermediate‐risk groups. A similar pattern was seen between the high‐intermediate‐risk and high‐risk groups. This trend was also observed in the PIT analysis.

### 3.6 Significance of the HPI as a prognostic factor

**FIGURE 4:**
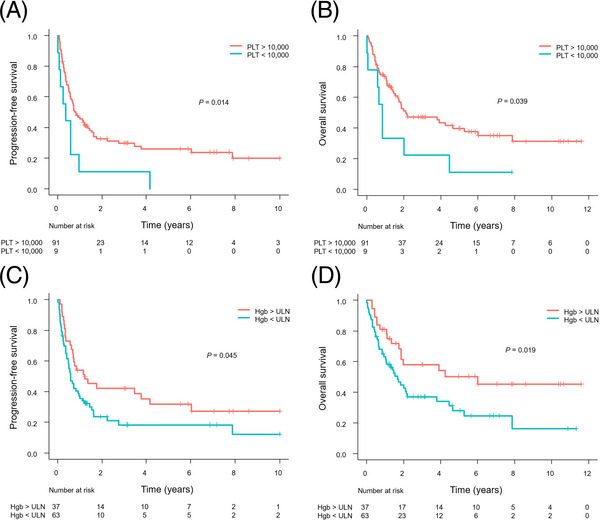
Kaplan–Meier analysis of progression‐free survival (PFS) (A) and overall survival (OS) (B) according to thrombocytopenia status. Kaplan–Meier analysis of PFS (C) and OS (D) according to anemia status.

**FIGURE 5:**
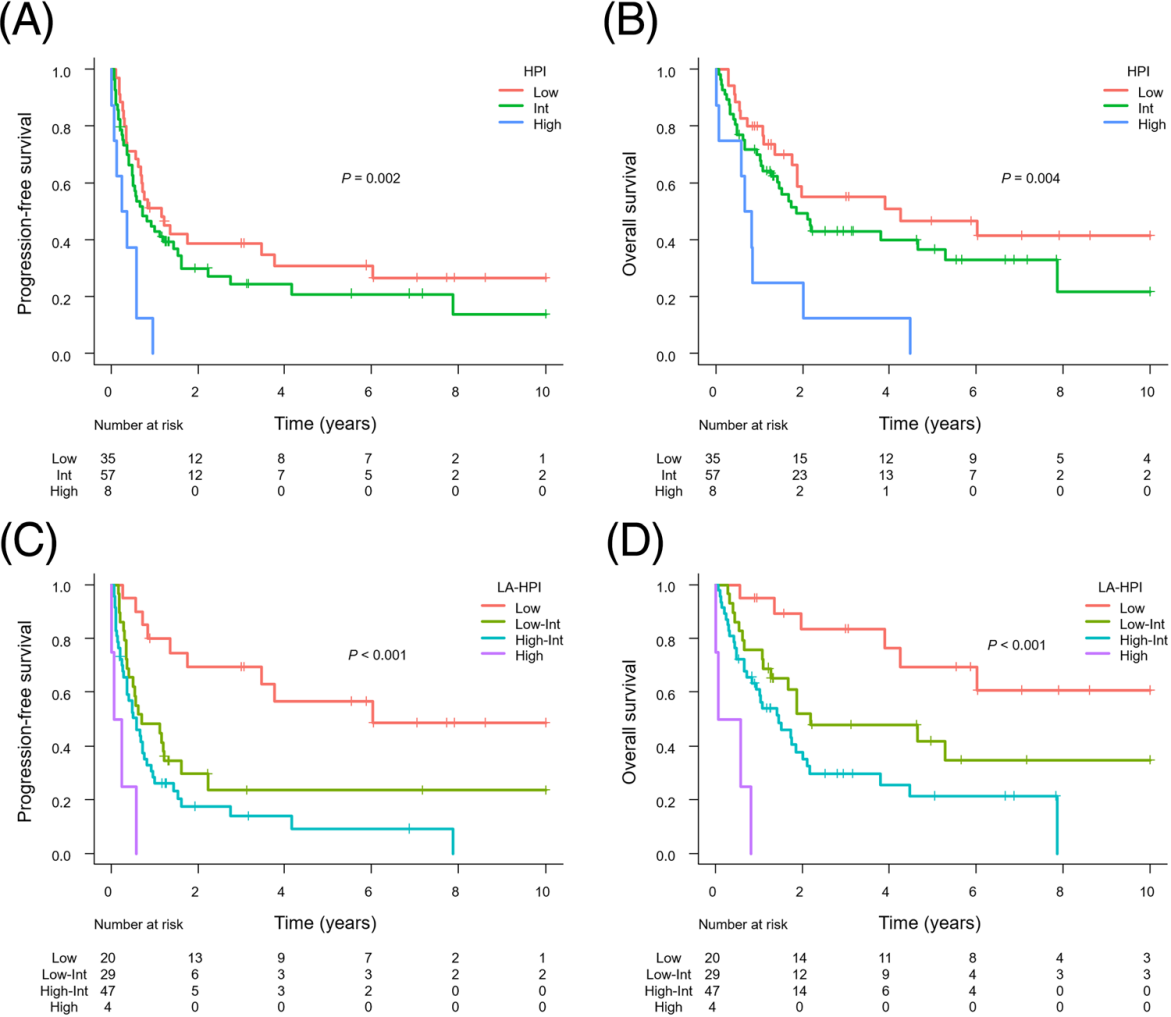
Kaplan–Meier analysis of progression‐free survival (PFS) (A) and overall survival (OS) (B) according to the hemoglobin‐platelet index (HPI). Kaplan–Meier analysis of PFS (C) and OS (D) according to the LA‐HPI.

Patients with thrombocytopenia had significantly worse PFS and OS than those without thrombocytopenia (Figure 4A,B). Similarly, patients with anemia had significantly worse PFS and OS than those without anemia (Figure 4C,D). According to the HPI, 35 (35%) patients were classified as low‐risk, 57 (57%) patients as intermediate risk, and eight (8.0%) patients as high risk. The HPI showed a significant association with PFS and OS (Figure 5A,B).

### 3.7 Proposal for a novel prognostic index incorporating the HPI, LDH, and albumin

**FIGURE 6:**
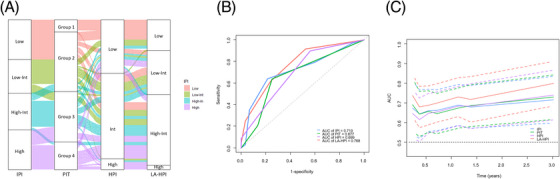
The alluvial plot shows the association of International Prognostic Index (IPI), Prognostic Index for PTCL‐unspecified (PIT), hemoglobin‐platelet index (HPI), and LA‐HPI in the entire cohort (A). The ROC curves according to 2‐year progression‐free survival (PFS) (B) and time‐dependent ROC curves for PFS (C).

**FIGURE 7:**
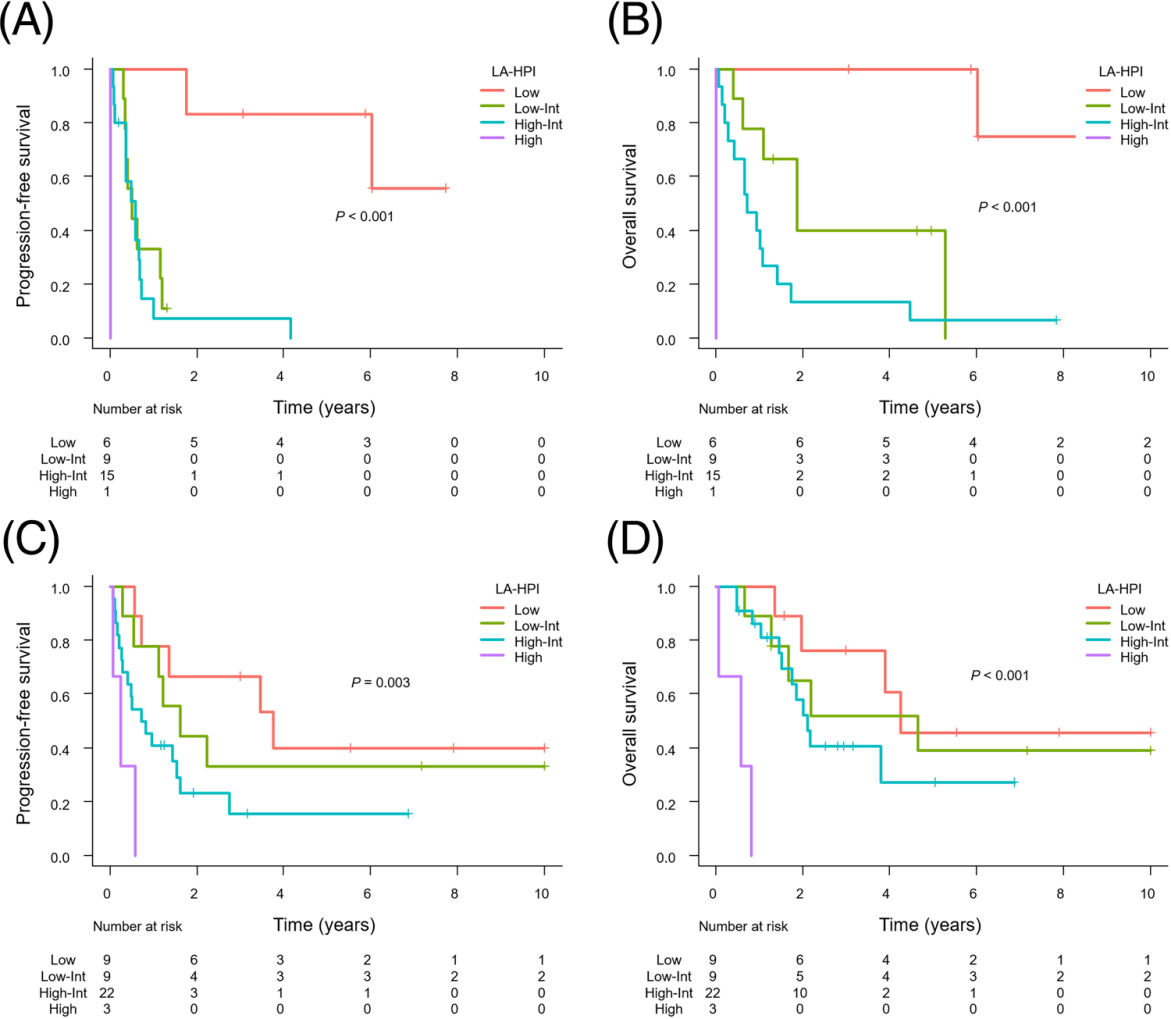
Kaplan–Meier analysis of progression‐free survival (PFS) (A) and overall survival (OS) (B) in patients with PTCL‐NOS according to the LA‐HPI. Kaplan–Meier analysis of PFS (C) and OS (D) in patients with AITL according to the LA‐HPI.

On multivariate analysis of PFS, elevated LDH and hypoalbuminemia were significantly associated with poor PFS. We therefore proposed a new prognostic model combining the HPI, LDH, and albumin, called the LA‐HPI. In this scoring system, patients are categorized as low risk (zero risk factors), low‐intermediate risk (one risk factors), high‐intermediate risk (two or three risk factors), or high risk (four risk factors) based on the number of risk factors. According to the LA‐HPI, 20 (20%) patients were low risk, 29 (29%) were low‐intermediate risk, 47 (47%) were high‐intermediate risk, and four (4.0%) were high risk. The PFS and OS differed significantly among the patients by the LA‐HPI score (Figure 5C,D). The alluvial plot provides a visual representation of the relationship between the IPI, PIT, HPI, and LA‐HPI in the entire cohort (Figure 6A). Then, we compared the predictive performance of these indices. The Harrell's C‐index of LA‐HPI for PFS was better than that of IPI, PIT, and HPI (0.662, 0.636, 0.629, and 0.575, respectively). Additionally, the predictive values of these prognostic indices were evaluated by the ROC curves (Figure 6B). According to the 2‐year PFS, the LA‐HPI exhibited a superior AUC compared to the IPI, PIT, and HPI (0.749, 0.699, 0.681, and 0.687, respectively). To track the changes in AUC among these models throughout the follow‐up period, time‐dependent ROC curves were also constructed. These curves revealed a consistent superiority of LA‐HPI over the IPI, PIT, and HPI throughout the entire observation period (Figure 6C). The LA‐HPI was able to distinguish the PFS and OS between the PTCL‐NOS (*n* = 31) (Figure 7A,B) and AITL (*n* = 43) groups (Figure 7C,D).

## 4 DISCUSSION

The present study investigated the prognostic value of the HPI in patients with PTCL and found the HPI to be an independent predictor of PFS and OS. We constructed a prognostic index by combining the HPI, LDH, and albumin, previously identified independent prognostic factors. The new prognosis score, based on parameters easily obtainable in routine clinical practice, was able to stratify patients by PFS more efficiently than other indices. To the best of our knowledge, this is the first study to focus on the clinical value of the HPI for PTCL.

The prognostic value of the HPI was originally investigated in DLBCL patients [19]. Thrombocytopenia was reportedly associated with a poor prognosis in patients with DLBCL and PTCL [16, 18, 25, 26, 27]. The presence of anemia was also associated with a poor prognosis in follicular lymphoma and is used regularly in clinical practice as an assessment item in the Follicular Lymphoma International Prognostic Index (FLIPI) and FLIPI2 [28, 29]. Anemia was also related to a poor prognosis in patients with DLBCL and PTCL [17, 25, 30]. Although the pathogenesis of thrombocytopenia and anemia in lymphoma is multifactorial and not completely understood, it is assumed to result from an autoimmune‐mediated process and involves the bone marrow and inflammatory factors [19, 31]. Based on these previous researches illustrating the prognostic significance of thrombocytopenia and anemia in patients with PTCL, we aimed to evaluate the prognostic utility of the HPI in this study.

In our cohort, the HPI was able to stratify the PFS and OS in patients with PTCL. Furthermore, we developed a more powerful prognostic index by combining HPI, LDH, and albumin. LDH is thought to reflect the tumor burden in patients with lymphoma, including PTCL, and is one of the variables in the IPI and PIT [32]. Hypoalbuminemia was also shown to be a significant risk factor for increased mortality in patients with PTCL [33, 34]. The high prognostic values of these factors may help enhance the predictive power of the LA‐HPI beyond that of the HPI.

AITL often manifests systematic symptoms associated with immune dysregulation and shows positive results on autoimmune tests [35]. In particular, autoimmune cytopenia can be seen as an initial presentation of AITL [36]. A previous study identified the presence of thrombocytopenia as an important, predictive factor for a poor prognosis in AITL [37]. In the present study, the LA‐HPI index had a statistically significant prognostic value in the subgroup of patients with AITL and was useful in predicting their clinical outcomes.

PTCL is a heterogeneous group comprising aggressive forms of NHL and has a poor outcome following anthracycline‐based chemotherapy, even when consolidation with ASCT is performed [4, 6, 7]. Frontline treatment with BV+CHP demonstrated superiority to CHOP in terms of a statistically significant improvement in PFS and OS in patients with CD30‐positive PTCL and had a good safety profile. However, a pivotal study demonstrated that the benefits of BV+CHP were limited to patients with IPI < 4 [8]. A phase II trial is currently underway to investigate the safety and efficacy of adding etoposide to BV+CHP followed by BV consolidation in patients with CD30‐positive PTCL [38]. On the other hand, patients who achieved complete response (CR) with CHOP did not always have a poor outcome without ASCT [5]. In the present study, most of the patients received CHOP‐based induction chemotherapy with or without BV, and only two patients underwent upfront ASCT. However, there was a proportion of patients who experienced long‐term survival or a cure after initial chemotherapy. Therefore, it is meaningful to assess the prognosis of patients before administering the initial treatment. Treatment strategies based on predicted prognosis have not yet been well‐established in PTCL. However, the HPI and LA‐HPI have demonstrated robust prognostic ability and are anticipated to be the basis of determining the treatment strategy in the future investigations.

Several recent studies focused on gene expression profiling and microenvironmental immune cell signatures in PTCL [39, 40, 41]. Although these studies have shed light on PTCL biology, which has led to improvements in the therapeutic approach, there is presently no definite evidence that these results have any direct bearing on clinical practice. Considering that most of the methods examined in these reports are expensive and require special equipment, prognostic and predictive markers that are inexpensive and easily available in daily practice are still needed. Because the hemoglobin level, platelet count, LDH, and albumin level can be easily and reproducibly measured in routine practice, the LA‐HPI deserves further investigation.

The present study has some limitations. First, it was a retrospective study enrolling a relatively small cohort and was conducted at a single center; thus, there are likely to be some biases, including selection bias. However, the latter was mitigated by including consecutive patients over a defined period. Second, the study population was heterogeneous with respect to various histological subtypes and treatment strategies, which might affect the robustness of our statistical interpretation. However, the LA‐HPI demonstrated good discrimination of survival outcomes in the subgroups of both AITL and PTCL‐NOS. Third, we were unable to explore the biological basis for the association between the HPI and LA‐HPI and the prognosis of PTCL patient, as previously demonstrated in the study examining the correlation between anemia/thrombocytopenia and interleukin‐6 production in DLBCL cells [19]. Finally, our study was conducted in a single cohort, potentially limiting the generalizability of our findings to other populations. We could not perform validation in independent cohorts; therefore, future studied are necessary to confirm the prognostic significance of the HPI and LA‐HPI in patients with PTCL. Despite these limitations, the data presented herein are the first to suggest that the HPI is an important prognostic factor in PTCL. Considering that the HPI and LA‐HPI consist of readily obtainable parameters, they are very useful indices for use in the standard clinical setting.

## 5 CONCLUSION

In conclusion, the HPI, based on anemia and thrombocytopenia values, was a significant prognostic factor of PTCL. Moreover, the LA‐HPI demonstrated good ability to discriminate survival outcomes in patients with PTCL using only four parameters that are easily available in routine clinical practice.

## AUTHOR CONTRIBUTIONS

Conceptualization: Tatsu Shimoyama. Investigation: Yu Yagi, Yusuke Kanemasa, Yuki Sasaki, Shunichi Okumura, Takako Watanabe, Kento Ishimine, Yudai Hayashi, Mano Mino, An Ohigashi, Yuka Morita, Taichi Tamura, Shohei Nakamura, Toshihiro Okuya, and Tatsu Shimoyama. Writing—original draft: Yu Yagi. Writing—review and editing: Yusuke Kanemasa.

## CONFLICT OF INTEREST STATEMENT

The authors declare they have no conflicts of interest.

## FUNDING INFORMATION

The authors received no specific funding for this work.

## ETHICS STATEMENT

The present, retrospective study was conducted in accordance with the Declaration of Helsinki and was approved by the Ethics Committee of Tokyo Metropolitan Cancer and Infectious Diseases Center, Komagome Hospital.

## CLINICAL TRIAL REGISTRATION

The authors have confirmed clinical trial registration is not needed for this submission.

## PATIENT CONSENT STATEMENT

Written informed consent was waived because this study used retrospective data obtained from the hospital medical records.

## Data Availability

The datasets generated during and/or analyzed during the current study are available from the corresponding author upon reasonable request.
